# Amniotic Fluid Stem Cell-Derived Extracellular Vesicles Counteract Steroid-Induced Osteoporosis In Vitro

**DOI:** 10.3390/ijms22010038

**Published:** 2020-12-22

**Authors:** Martina Gatti, Francesca Beretti, Manuela Zavatti, Emma Bertucci, Soraia Ribeiro Luz, Carla Palumbo, Tullia Maraldi

**Affiliations:** 1 Department of Biomedical, Metabolic and Neural Sciences, University of Modena and Reggio Emilia, 41125 Modena, Italy; martina.gatti@unimore.it (M.G.); francesca.beretti@unimore.it (F.B.); manuela.zavatti@unimore.it (M.Z.); sribeiro@unimore.it (S.R.L.); carla.palumbo@unimore.it (C.P.); 2 Department of Medical and Surgical Sciences for Mothers, Children and Adults, University of Modena and Reggio Emilia, Azienda Ospedaliero Universitaria Policlinico, Via Del Pozzo 71, 41124 Modena, Italy; emma.bertucci@unimore.it

**Keywords:** AFSC, extracellular vesicles, oxidative stress, osteoporosis

## Abstract

Background—Osteoporosis is characterized by defects in both quality and quantity of bone tissue, which imply high susceptibility to fractures with limitations of autonomy. Current therapies for osteoporosis are mostly concentrated on how to inhibit bone resorption but give serious adverse effects. Therefore, more effective and safer therapies are needed that even encourage bone formation. Here we examined the effect of extracellular vesicles secreted by human amniotic fluid stem cells (AFSC) (AFSC-EV) on a model of osteoporosis in vitro. Methods—human AFSC-EV were added to the culture medium of a human pre-osteoblast cell line (HOB) induced to differentiate, and then treated with dexamethasone as osteoporosis inducer. Aspects of differentiation and viability were assessed by immunofluorescence, Western blot, mass spectrometry, and histological assays. Since steroids induce oxidative stress, the levels of reactive oxygen species and of redox related proteins were evaluated. Results—AFSC-EV were able to ameliorate the differentiation ability of HOB both in the case of pre-osteoblasts and when the differentiation process was affected by dexamethasone. Moreover, the viability was increased and parallelly apoptotic markers were reduced. The presence of EV positively modulated the redox unbalance due to dexamethasone. Conclusion—these findings demonstrated that EV from hAFSC have the ability to recover precursor cell potential and delay local bone loss in steroid-related osteoporosis.

## 1. Introduction

Osteoporosis, involving increased bone porosity, is a common systemic skeletal disorder characterized by low bone mass and both structural and micro architectural deterioration of bone tissue, leading to bone fragility and an increased risk of fractures of the hip, spine, and wrist [1]. Clinically, this complex skeletal disorder can be divided into two types: Primary and secondary osteoporosis. The first is a systemic disease closely related to post-menopausal estrogen deficiency, while the second represents a particular set of dysregulations caused by different conditions or treatments [2]. The second is also known as senile or age-related osteoporosis and can occur in both men and women with age. Finally, secondary osteoporosis refers to when the disorder is present as a consequence of an adverse response to a medication, change in physical activity, or another medical condition. Common examples of this type include glucocorticoid- and immobilization-induced osteoporosis [3]. Moreover, advancing age causes age-related skeletal alterations even aggravated by the need to use treatments affecting bone mass, such as glucocorticoids (GC). Thus, glucocorticoid-induced osteoporosis (GIOP) is the primary cause of secondary osteoporosis but they are widely used in treatment of different diseases [4]. Long-term GC treatment inhibits the differentiation and activity of osteoblasts and osteocytes, which finally leads to a GIOP [5].

Overall, the key issue related to bone loss is thought to be the imbalance of remodeling cycle, implying the alternating and balanced processes of bone formation and bone erosion [6]. Indeed, in osteoporotic patients, there are deficiencies in resident stem cells, namely bone mesenchymal stem cells (BMSCs), since disorders in BMSCs proliferation and osteoblastic differentiation abilities occur and can ultimately give rise to osteoporosis. Therefore, resident BMSCs are closely related to the occurrence of osteoporosis, being a key part of the homeostatic machinery of bone turnover [7,8].

In the light of these considerations, several authors recently investigated in vivo and in vitro the potential of secretome and of secreted extracellular vesicles (EV) from mesenchymal stem cells in recovering bone precursor cell ability to overcome senescence and apoptosis therefore delaying bone loss, as reviewed in [9,10,11].

In vitro studies employed adult stem cells obtained from rat bone marrow [12] or adipose mesenchymal stem cells [13]. The co-culture of MSC-EV with bone cell lines gave encouraging results. On the other hand, as osteoporosis in vivo models, ovariectomized rats [14,15] or aged animals [8,16] were used. Interestingly, in these studies, the sources of human MSC were formerly from adult tissues [14] or, more recently, from younger tissues, such as human exfoliated deciduous teeth [15] and umbilical cord [8,16].

Notably, increased osteocyte apoptosis may occur during aging because cells suffer from pathological metabolic processes that lead to the rise of oxidative stress, lack of hormones, and disruption of blood flow [17]. Thus, levels of reactive oxygen species (ROS) should also be investigated in this context. Indeed, BMSCs from aged rats display higher levels of ROS than those from young rats [8] Moreover, the treatment with the GC dexamethasone (DEXA) increases the intracellular ROS level too, indicating that DEXA-derived ROS can lead to the pro-apoptotic effect on osteoblasts. Furthermore, DEXA has shown activity in regulating osteoblast differentiation [18].

Recently a strong body of studies claims that autophagy, as a cell survival pathway, plays a vital role in maintaining bone homeostasis and changes in this pathway are to some extent associated with osteoporosis [19]. In bone, an age-related decreased autophagic activity was recently described in rat osteocytes: In particular, a decreased expression of LC3-II, BECN1 and ULK-1 was observed with age while p62 expression and apoptosis increase in osteocytes [20]. Hence, oxidative stress resulting from autophagy decline might be one of the factors involved in age-associated bone loss. However, oxidative stress induced by ovariectomy was recently shown to be associated with an increased in osteocyte autophagic activity [20]. In addition, it was observed that, to respond to this oxidative stress, autophagy was upregulated to protect osteoblasts from apoptosis [19], highlighting the possible correlation between the dose of dexamethasone and the induction of autophagy via intracellular ROS.

Amniotic fluid stem cells (AFSCs) are a feasible variety of MSC for experimentation purposes, owing to their pluri/multi-potency due to the early stage of their collection, abundance, and minimal ethical considerations. We have previously demonstrated that AFSC-extracellular vesicles contain anti-inflammatory molecules, namely HFG, TGFβ, and pentraxin 3, that showed a therapeutic effect on osteoarthritis [21], and antioxidant enzyme, such as SOD1 [22]. The application of AFSC-EV could be effective in osteoporosis since even osteoclast activity can be affected by these anti-inflammatory and antioxidant molecules. Indeed, it was found that ROS actively take place in osteoclast differentiation by acting as secondary signaling molecules [23].

The present study aims to explore the effects of EV, enriched in exosomes, derived from human AFSC, therefore from very young MSC, on osteoblasts apoptosis. Pre-osteoblasts from human origin (HOB), induced or not toward the differentiation process, and treated with DEXA for 24 h to induce the osteoporosis feature, were the in vitro model used for this study (see Supplementary Figure S1). Thereafter the effects of the glucocorticoid on viability, apoptosis, and differentiation, have been examined. Furthermore, the correlation between oxidative, autophagic, and osteoporotic status has been investigated, since these are the most debated aspects [20]. The paracrine effect of AFSC, mediated by EV, has been evaluated for all these processes both in pre-osteoblasts and in differentiated cells exposed to DEXA, in order to dissect if their protecting ability could be successful in osteoporosis disorders.

## 2. Results

### 2.1. Characterization of AFSC Extracellular Vesicles

Western blot (WB) characterization of AFSC-EV, obtained from three donors, revealed that, even if EV have been harvested twice for each sample (first and second collection), all the collections maintained similar levels of exosome markers (CD9 and Rab5), and of immunomodulating proteins as well (TGFβ, IDO, HGF), as shown in Supplementary Figure S2. These data allowed us to manage a double quantity of EV for each donor, by using a mixture of first and second collected EV even maintaining the individual diversity.

### 2.2. Effect of AFSC-EV on Viability and Apoptotic Process Modulated by Dexamethasone

**Figure 1:**
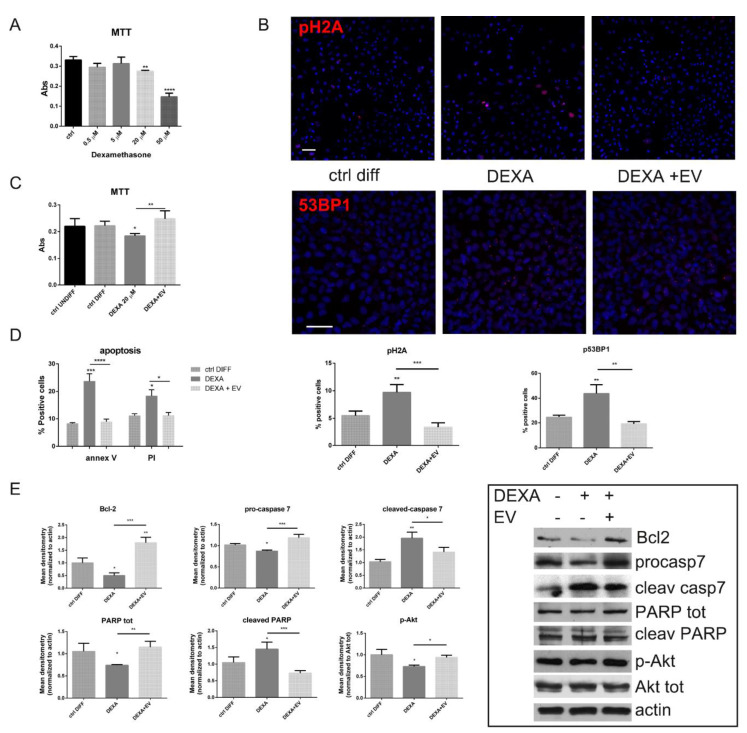
Effect of extracellular vesicles secreted by human amniotic fluid stem cells (AFSC-EV) supplementation on cell both viability and apoptotic pathway of human pre-osteoblast cells (HOB) treated or not with Dexamethasone. (**A**)—Graph showing MTT assay of HOB cells treated with increasing concentrations of dexamethasone (DEXA). ** *p* value < 0.01; **** *p* value < 0.0001. (**B**)—Representative images with 4’,6-diamidino-2-phenylindole (DAPI) (blue) and pH2A (red) or 53BP1 (red) signals of HOB cells incubated or not with DEXA and extracellular vesicles (EV) are shown. Scale bar = 25 µm. Percentage of positive cells visualized in 5 fields for each condition are shown in the graphs below. ** *p* value < 0.01; *** *p* value < 0.001. (**C**)—Graph showing MTT assay of HOB cells pre-treated with EV and then exposed to 20 µM DEXA. * *p* value < 0.05; ** *p* value < 0.01. (**D**)—Graph showing the percentage of positive cells to Annexin V/Propidium Iodide assay, as described in the M&M section. * *p* value < 0.05; *** *p* value < 0.001; **** *p* value < 0.0001. (**E**)—WB analysis of total lysate of HOB cells treated or not with AFSC-EV in the presence of DEXA, then revealed with anti-Bcl-2, anti-caspase 7, to detect the procaspase and the cleaved form, anti-PARP, to detect PARP total and its cleaved form, anti-pSer473Akt, and anti-Akt total antibodies. The graphs represent the mean ± SD of densitometric analysis of three experiments, normalized to actin values. * *p* value < 0.05; ** *p* value < 0.01; *** *p* value < 0.001.

MTT assay with increasing concentrations of dexamethasone showed that HOB viability was significantly decreased by 60% by 50 µM DEXA treatment, while, albeit to a lesser extent, starting from 20 µM, this parameter was significantly affected (Figure 1A). Based on this result, we chose to perform all the following experiments with 20 µM DEXA.

Since the exposure with 20 µM DEXA induced a partial viability decrease, we investigated DNA damage, analyzing 53BP1, a binding protein of p53, a well-known DNA damage response (DDR) factor, and the phosphorylation of the histone variant H2A.X that initiates the activation of the DDR pathway. As shown by immunofluorescence (Figure 1B), the 53BP1 and pH2A labelling increased in the presence of DEXA, while EV pre-treatment avoided these effects. Indeed, DNA damage leads to the induction of a p53 response, however the ultimate response to p53 can be quite different, ranging from a reversible cell cycle arrest to the induction of a number of irreversible responses, such as cell death or senescence [24].

The analysis of apoptosis by annexin V and propidium iodide (PI) demonstrated the involvement of apoptotic pathway, as expected (Figure 1D). However, the presence of EV significantly restored cell viability (Figure 1C) and reduced annexin V and PI staining (Figure 1D). Consistently with this result, our immunoblotting data further demonstrated an increased level of Bcl-2, an anti-apoptotic marker, in HOB-EV cells (Figure 1E). Moreover, the cleavages of PARP and caspase 7, significantly higher in DEXA-treated cells, compared to control cells, were reduced by the EV- treatment. In parallel, EV triggered an activation of Akt, a key signaling molecule for survival, indicated by its higher phosphorylation in Ser 473, normalized to Akt total.

These results suggest that the decreased cell viability associated with the activation of apoptotic pathway induced by DEXA can be contained by AFSC-EV.

### 2.3. Effect of AFSC-EV on Autophagic Pathway Affected by Dexamethasone

Recently a strong cohort of studies claims that autophagy, as a cell survival pathway, plays a vital role in maintaining bone homeostasis and changes in this pathway are to some extent associated with osteoporosis [19].

**Figure 2:**
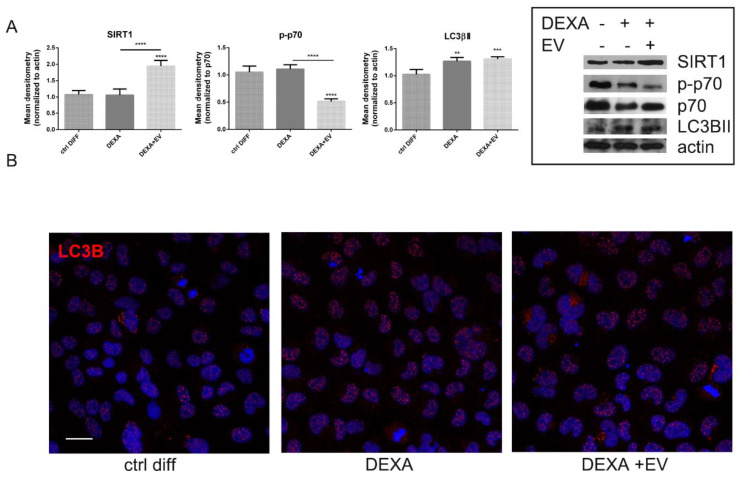
Effect of AFSC-EV supplementation on autophagic pathway of HOB treated or not with Dexamethasone. (**A**)—Western blot analysis of total lysate of HOB cells treated or not with AFSC-EV in the presence of DEXA, then revealed with anti-SIRT1, anti-p-p70, anti-p70, and anti-LC3β antibodies. The graphs represent the mean ± SD of densitometric analysis of three experiments, normalized to actin values. ** *p* value < 0.01; *** *p* value < 0.001; **** *p* value < 0.0001. (**B**)—Representative images with DAPI (blue) and LC3β (red) signals of HOB cells incubated or not with DEXA and EV are shown. Scale bar = 10 µm.

Interestingly, autophagic pathway was also positively modulated through AFSC-EV content, as demonstrated by the increased levels of silent information regulator of transcription1 (SIRT1) (Figure 2A), a NAD-dependent class III histone and non-histone protein deacetylase that promotes autophagy. Then, the phosphorylation of p70 and the level of LC3β, markers of autophagy, even if in opposite manner, were investigated. The active form of LC3β, LC3 βII, was higher during osteoporosis induction in WB analysis (Figure 2A), but the localization of the staining was still nuclear (Figure 2B). However, EV- treatment was able to contrast the phosphorylation of p70 (Figure 2A) and to increase LC3β localization into the cytoplasm, as shown in IF images (Figure 2B), translocation induced by a deacetylation process by SIRT1 [25].

### 2.4. ROS Modulation by AFSC-EV in Osteoblasts Treated with Dexamethasone

**Figure 3:**
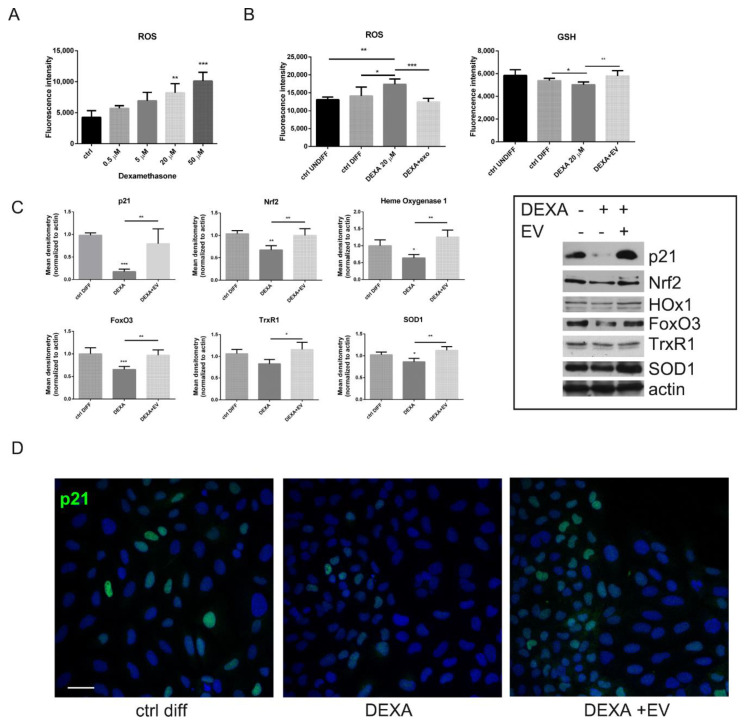
Effect of AFCS-EV supplementation on reactive oxygen species (ROS) modulation in HOB treated or not with Dexamethasone. (**A)**—Graph showing ROS assay of HOB cells treated with increasing concentration of DEXA. ** *p* value < 0.01; *** *p* value < 0.001. (**B)**—ROS and GSH content was measured with fluorescent probes, after AFSC-EV exposure and DEXA treatment. * *p* value < 0.05; ** *p* value < 0.01; *** *p* value < 0.001. (**C)**—Western blot analysis of total lysate of HOB, treated or not with AFSC-EV, then exposed to DEXA. Immunoblot were performed with the indicated primary antibodies. The graphs represent the mean ± SD of densitometric analysis of three experiments, normalized to actin values. * *p* value < 0.05; ** *p* value < 0.01; *** *p* value < 0.001. (**D)**—Representative images with DAPI (blue) and p21 (green) signals of HOB cells incubated or not with DEXA and EV are shown. Scale bar = 10 µm.

The presence of DEXA induced a dose-dependent increase of ROS (Figure 3A), as expected: HOB displayed a higher ROS level compared to untreated cells, in a significant manner only starting from 20 µM DEXA. However, the exposure to AFSC-EV reduced the ROS content, meanwhile allowing the increase in reduced glutathione (GSH) level (Figure 3B).

Then, we investigated the expression of proteins related to ROS modulation: WB analysis (Figure 3C) and IF images (Figure 3D) demonstrated that EV-treated HOB can induce an increase in p21, dramatically reduced in DEXA-treated cells. Actually, p21 plays a role in controlling ROS by positively regulating transcriptional activity of Nrf2 [26]. Indeed, the redox sensitive transcription factors Nrf2 and FoxO3 were affected by DEXA but increased in EV samples, as shown in Figure 3C. Therefore, antioxidant enzymes regulated by these transcription factors, such as superoxide dismutase 1 (SOD1), heme oxygenase 1 (HOx1), and thioredoxin reductase (TrxR1), were present in greater quantities in HOB + EV cells rather than in DEXA-treated cells.

### 2.5. Effect of AFSC-EV on Osteogenic Differentiation Reduced by Dexamethasone

**Figure 4:**
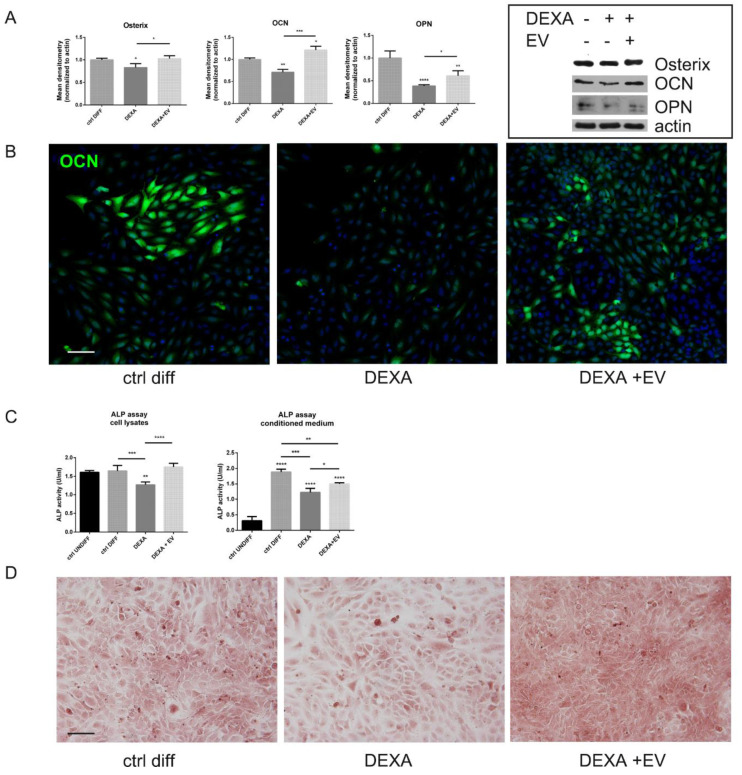
Effect of AFSC-EV supplementation on osteogenic differentiation markers of HOB treated or not with Dexamethasone. (**A**)—Western blot analysis of total lysate of differentiated HOB cells treated or not with AFSC-EV and DEXA, then revealed with the indicated antibodies. The graphs represent the mean ± SD of densitometric analysis of three experiments, normalized to actin values. * *p* value < 0.05; ** *p* value < 0.01; *** *p* value < 0.001; **** *p* value < 0.0001. (**B**)—Representative images showing DAPI (blue) and osteocalcin (OCN) (green) signals of HOB cells incubated or not with EV and DEXA. Scale bar= 30 μm. (**C**)—ALP assay performed on total lysate or on conditioned medium, as reported in M&M section, of HOB treated or not with AFSC-EV and DEXA. * *p* value < 0.05; ** *p* value < 0.01; *** *p* value < 0.001; **** *p* value < 0.0001. (**D**)—Representative images of Alizarin Red staining of HOB cells incubated or not with AFSC-EV and DEXA are shown. Scale bar = 50 μm.

HOB cells, treated with DEXA even at the end of the differentiation protocol (see the experimental scheme in Supplementary Figure S1), showed a decrease in the expression of osteogenic markers, such as osterix (OSX), osteocalcin (OCN), and osteopontin (OPN), as expected (Figure 4A,B). Moreover, the assay of intracellular and secreted alkaline phosphatase (ALP) (Figure 4C) and the staining with Alizarin Red to evaluate calcium deposits (Figure 4D) also confirmed that the bone matrix deposition was affected by DEXA. Interestingly, the preincubation with AFSC-EV reversed these negative effects, significantly restoring the levels of all these markers.

### 2.6. Effect of AFSC-EV on Undifferentiated Pre-Osteoblasts

The above-described effect of EV on differentiated HOB was evident even in undifferentiated HOB. Indeed, four-day exposure to AFSC-EV induced in the preosteoblast cell line a differentiation process towards the osteoblast feature.

**Figure 5:**
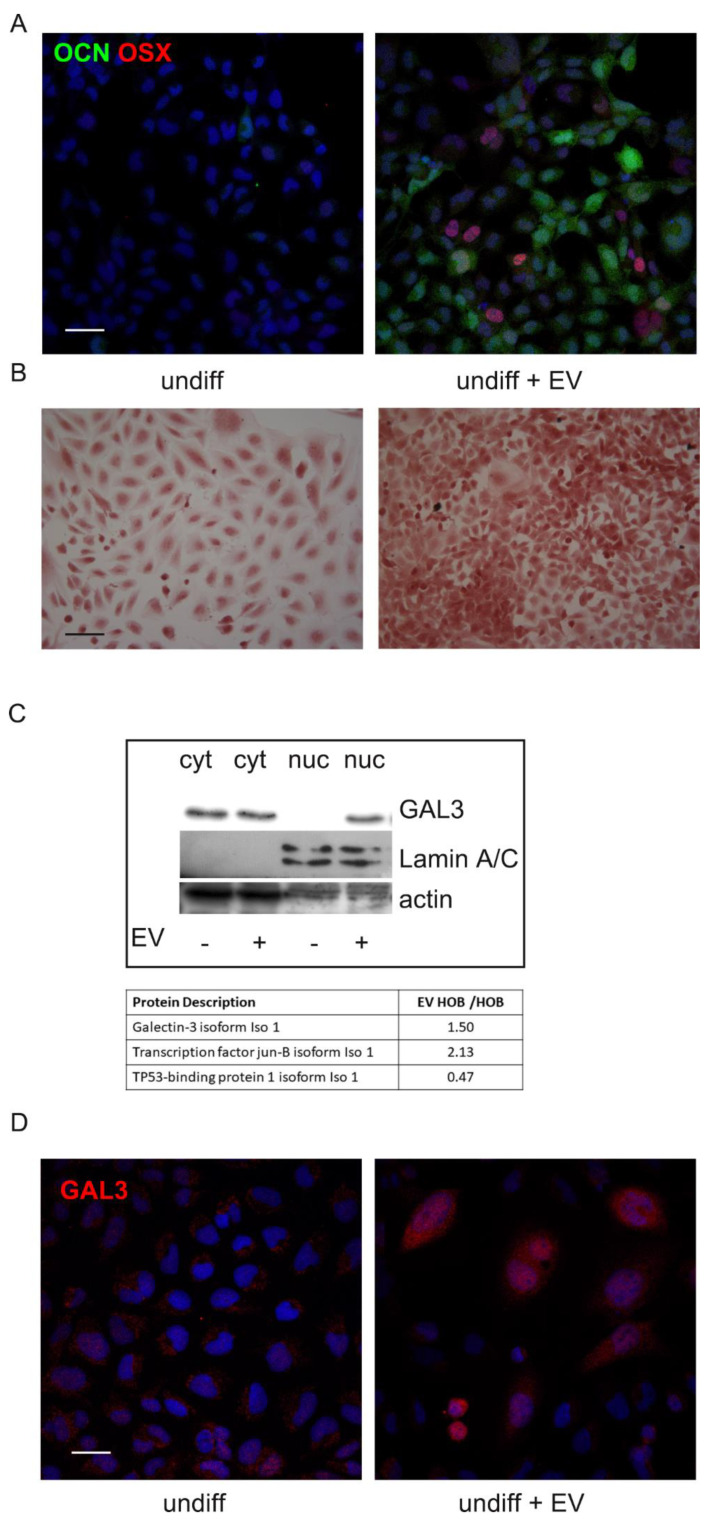
Effect of AFSC-EV supplementation on osteogenic differentiation process of undifferentiated HOB. (**A**)—Representative images showing DAPI (blue) and OCN (green) and osterix (OSX) (red) signals of undifferentiated HOB cells incubated or not with EV. Scale bar= 30 μm. (**B**)—Representative images of Alizarin Red staining of HOB cells incubated or not with AFSC-EV are shown. Scale bar= 50 μm. (**C**)—Table shows mass spectrometry semi-quantitative analysis of nuclear proteins identified in undifferentiated HOB treated or not with EV. The ratio between EV-treated HOB and untreated is shown for the proteins identified in both the samples and involved in osteogenic differentiation process. WB analysis of nuclear (nuc) and cytosolic (cyt) lysates of undifferentiated HOB cells treated or not with AFSC-EV, then revealed with anti-Galectin3, anti-lamin A/C, as nuclear marker, and anti-actin, as a loading control. (**D**)—Representative images showing DAPI (blue) and Galectin-3 (red) signals of undifferentiated HOB cells incubated or not with EV. Scale bar = 10 μm.

In fact, immunofluorescence analysis showed that the presence of osteoblast markers, such as the transcription factor osterix and the secreted protein osteocalcin, was increased in EV-treated cells (Figure 5A). Moreover, Alizarin Red staining definitely confirmed the increase in deposition of calcified extracellular matrix due to EV (Figure 5B).

In order to investigate the mechanism underpinned by AFSC-EV for the induction of the osteogenic differentiation process, nuclear cell extracts of HOB were obtained for proteomic experiments. Mass spectrometry analysis revealed that in the presence of EV derived from AFSC, HOB cell line expressed more osteogenic differentiation related proteins compared to the untreated cells and proteins inhibitors of bone differentiation were downregulated (Table in Figure 5C). Indeed, among the nuclear proteins identified by MS, we focused our attention only on the proteins playing a role in osteogenic differentiation, as reported in the literature [27,28].

Moreover, since we previously found in the AFSC-EV proteome analysis [29] the presence of Galectin-3 binding protein, the levels of Galectin-3, as osteoblast modulating protein [30], was investigated. Western blot analysis and IF images demonstrated that the amount of Galectin-3 is dramatically higher in nuclei of HOB exposed to EV (Figure 5C,D), as suggested by proteomic analysis.

## 3. Discussion

Oxidative stress plays a role in the osteoporosis process as it affects bone homeostasis and remodeling, by inducing apoptosis and interfering with osteoblast autophagy [31]. Glucocorticoids, such as DEXA, depending on the concentration [18,31,32], can exert a negative effect on bone tissue by provoking intracellular ROS generation that is linked to cell death, defect on cell differentiation, and autophagic pathways. In this study, the treatment with DEXA induced a ROS rise, a decrease in antioxidant defenses, and a parallel boost of apoptosis and of DNA damage. In this scenario, we applied EV derived from gestational stem cells since have been previously demonstrated, even by our lab, that antioxidant enzymes are included into this cargo [21,33]. Actually, here we demonstrated positive results on viability, redox level, autophagy, and differentiation that can be due to the ROS modulation by EV. However, the EV content is very complex so a multifactorial effect cannot be excluded. Therefore, we focused the attention on a possible parallel mechanism that triggered osteogenic differentiation, a crucial process to avoid osteoporosis.

Considering the EV role on redox modulation, interestingly we noticed that the presence of EV induced a huge increase of the SIRT1 level. Moreover, it has been reported that SIRT1 can play a role on the skeleton, at least in part, via cells of the osteoblast lineage. Indeed, deletion of Sirt1 in adult mice decreases cortical bone mass, indicating that the skeletal effects of Sirt1 are not restricted to development and growth [34]. Furthermore, recent studies have demonstrated that SIRT1 is a potent intracellular inhibitor of oxidative stress, inflammatory responses, and inducer of autophagic process as well [34]. Moreover, one of the major targets of Sirtuins are the FoxO family of transcription factors. FoxOs play a major role in the adaption of cells to a variety of stressors such as oxidative stress [34]. SIRT1 can deacetylate the FoxO factor, i.e., FoxO1, FoxO3a, and FoxO4, and subsequently stimulates the expression of antioxidant enzymes, such as catalase, SOD, and Trx, and, via an auto-feedback loop, also potentiates SIRT1 expression. Consistently, here we observed an increase of FoxO3 and of TrxR. Notably, SIRT1 is a potent inducer of autophagy and in addition to the direct control of autophagosome formation, SIRT1 can also regulate autophagy indirectly via FoxO signaling. Indeed, FoxO1 and FoxO3 can also promote autophagy, a process that declines with bone aging and is disturbed in several age-related disease [35]. Western blot analysis indicated that the level of phosphorylated p70 S6 kinase (p-p70 S6 kinase), which is a substrate protein in the mTOR pathway, and the ratio of p-p70 S6 kinase/p70 S6 kinase significantly decreased following EV exposure, suggesting a positive effect on autophagy [36]. Meanwhile, our results showed that LC3B, even though increased in DEXA-treated cells, moved to the cytoplasm as puncta (autophagosomes) only when exposed to EV. Deacetylation of LC3 by SIRT1 allows LC3 to interact with the nuclear protein DOR and return to the cytoplasm with DOR, where it is able to bind autophagy factors [25]. Altogether, these data confirm the pro-autophagic effect of AFSC-EV in DEXA-treated HOB cells, maybe through a redox- dependent regulation of SIRT1.

Beside an interplay between SIRT1 and FoxO, it has to be highlighted that the redox sensitive/modulating transcription factor Nrf2 is regulated by SIRT1 as well: Indeed, SIRT1 promotes the deacetylation of Nrf2, improving the stability of this transcription factor [37]. In line with such evidences, here we showed an increase of Nrf2 and of heme oxygenase 1 and superoxide dismutase 1, two target genes of Nrf2, after EV exposure. Another indirect way of Nrf2 induction is played by p21, that suppresses G1 to S cell cycle transition and is in turn activated by FoxO3a [38]. A SIRT1/FoxO3a-dependent cell regulatory function that has been linked to oxidative stress can be an important self-defense mechanism to detoxifying harmful reactive molecules [39]. Furthermore, it has been proposed that p21 serves a protective function, preventing apoptosis in some circumstances. It is largely accepted that p21 is a p53-regulated gene and since p53 has been tightly linked to apoptosis, it could be logical to assume that being a downstream effector of p53, p21 is part of this process. However, p21 expression is important for the growth arrest that occurs following stress, since this is a necessary process to allow the cell time to repair damage to its DNA [40].

The tumor suppressor p53 and its homologues, p63 and p73, play a pivotal role in the regulation of the DNA damage response, cellular homeostasis, development, aging, and metabolism. They have been implicated in apoptosis since the combined loss of p63 and p73 results in the failure of cells containing functional p53 to undergo apoptosis in response to DNA damage [41]. Moreover, p53 family as a whole, including p63 and p73, collaborate in controlling autophagy [42].

About this kind of damage, we monitored the staining with p53 binding protein 1 and pH2A, since 53BP1 plays a role early in the DNA damage response pathway and acts downstream of H2AX [43]. The EV incubation prevented the increase of these markers induced by DEXA, protecting HOB from DNA damage. Furthermore, all the apoptosis markers were positively modulated by EV pretreatment.

Phosphorylation at serine 46 has also been linked to the ability of p53 to repress expression of Galectin-3, an anti-apoptotic protein that can protect from p53-induced death [44]. Interestingly, we previously found into AFSC-EV the presence of Galectin-3 binding protein with a high score [31]. Moreover, it has been demonstrated that Galectin-3 plays a critical role in osteoblast differentiation [32]. Actually, here we showed the increase of Galectin-3 into the nuclei of HOB treated with EV (Figure 5C), modulating the splicing patterns of several genes [45,46]. Indeed, thanks to a proteomic approach, we analyzed the protein pattern promoted into the nuclei by EV presence. Notably, several genes, typical of osteogenic differentiation, were identified only in EV-treated samples (data not shown). Here we decided to discuss only the modulation of proteins present in both samples. Among these, AP-1 complex beta1, that interacts with c-Jun (more expressed in EV HOB), composes a transcription factor highly expressed in proliferating osteoprogenitors that promotes bone formation. Direct targets of AP-1 in osteoblasts include osteocalcin, bone sialoproteins, and alkaline phosphatase promoters [47]. On the other hand, negative regulator of osteoblast differentiation, such as TP53, whose pathway activation was correlated with subsequent cell apoptosis, which could impact development, particularly ossification [48], was less expressed in EV sample, compared to untreated HOB.

All these considerations can justify the pro-differentiation effect of AFSC-EV observed both in undifferentiated and under differentiation HOB cells as well. Indeed, the inhibitory effect of DEXA on osteogenesis was shown by the reduction of cell viability, decrease of ALP activity, and suppression of osteogenesis-related protein expression including ALP, OPN, and OCN.

In conclusion, the ability of AFSC-EV to contrast the negative effect of DEXA even in osteoblast differentiation suggests their possible role in the promotion of this process, but, more importantly, EV have demonstrated to counteract all the osteoporosis signs observed in HOB treated with DEXA.

## 4. Materials and Methods

### 4.1. Amniotic Fluid Collection

The AFSCs were obtained from amniotic fluids collected from 3 healthy pregnant women at the 16th week of gestation who underwent amniocentesis for maternal request (not for foetal anomalies) at the Unit of Obstetrics & Gynaecology, IRCCS—ASMN of Reggio Emilia and at the Policlinico Hospital of Modena (Italy). Amniocentesis were performed under continuous ultrasound guidance, in a sterile field, with 23-Gauge needles. The risks related to the procedure and the purpose of the study were explained to all patients before the invasive procedure and the ob-gyn specialist collected a signed consent before starting the exam (protocol 2015/0004362 of 02.24.2015 and protocol 360/2017 dated 12.15.2017 approved by Area Vasta Emilia Nord). For this study, supernumerary (unused) flask of AF cells, cultured in the Laboratory of Genetics of TEST Lab (Modena, Italy) for 2 weeks, were used.

### 4.2. Amniotic Fluid Stem Cell Isolation and Culture

AFSCs were isolated as previously described [49]. Human amniocentesis cultures were harvested by trypsinization and subjected to c-kit immunoselection by MACS technology (Miltenyi Biotec, Germany). AFSCs were subcultured routinely at 1:3 dilution and not allowed to grow beyond the 70% of confluence. AFSCs were grown in culture medium (αMEM) supplemented with 20% foetal bovine serum (FBS), 2 mM L-glutamine, 100 U/mL penicillin, and 100 μg/mL streptomycin (all from EuroClone Spa, Milano, Italy).

### 4.3. Extracellular Vesicle Isolation from Conditioned Medium

AFSCs were grown in 75 cm^2^ flask until subconfluence (around 1 ×10^6^ cells). Before extracellular vesicle extraction, the cells were maintained for 4 days in 10 mL culture medium deprived of FBS in order to exclude the contamination by extracellular vesicles comprised into FBS solution. The secreted part of conditioned medium (CM) was then concentrated up to 2 mL by using Centrifugal Filter Units with 3K cutoff (Merk Millipore, MA, USA) [29]. Then, the concentrated CM was treated with Total Exosome Isolation solution from cell culture media (Invitrogen, Life Technologies, CA. USA), according to manufacturer’s instructions. The pellet, enriched in exosomes although it can be not a pure extraction, was collected and quantified by Bradford method. To obtain a sample for Western blot analysis, the pellet was re-suspended in lysis buffer. We previously showed morphological characterization of extracellular vesicles by electron microscopy [29].

### 4.4. HOB Culture and Treatments

Human pre-osteoblast cells (HOB) were grown in culture medium (αMEM) supplemented with 10% FBS, 2 mM L-glutamine, 100 U/mL penicillin, and 100 μg/mL streptomycin (all from EuroClone Spa, Milano, Italy), in a humidified atmosphere with 5% CO_2_ at 37 °C. Osteogenic differentiation was obtained after 1 week of culture in a differentiation medium composed by αMEM supplemented with 1% FBS, 2 mM L-glutamine, 100 U/mL penicillin, and 100 μg/mL streptomycin (all from EuroClone Spa, Milano, Italy), 100 µM 2P-ascorbic acid, and 5 mM β-glycerophosphate (all from Sigma-Aldrich, St Louis, MO, USA).

One day after seeding, culture medium was replaced with differentiation medium (5 days) and after 24 h of differentiation extracellular vesicles (EV) were added for 4 days. Dexamethasone (Sigma Aldrich, St Louis, MO, USA) treatment was added at the end of differentiation, for 24 h at the concentration of 0.5, 5, 20, and 50 µM (Supplementary Figure S1).

### 4.5. MTT Assay

HOB were seeded in 96-well plates in 100 μL of a culture medium, 4 replicates for each condition, at the density of 500 cells/well. Cells were then differentiated and treated with EV for 4 days, as previously described. Dexamethasone (Sigma Aldrich, St Louis, MO, USA) treatment was performed after the EV exposure for 24 h at the concentration of 0.5, 5, 20, and 50 µM. At the end of experiment, 0.5 mg/mL MTT (Sigma Aldrich, St Louis, MO, USA) was added and incubated for 3 h at 37 °C. After incubation, the medium was removed and acidified isopropanol (Carlo Erba, Milan, Italy) was added to solubilize the formazan salts [50]. The absorbance was measured at 570 nm using a microplate spectrophotometer (Appliskan, Thermo-Fisher Scientific, Vantaa, Finland).

### 4.6. ROS and Glutathione Detection

To evaluate intracellular ROS levels, dichlorodihydrofluorescein diacetate (DCFH-DA, Sigma Aldrich, St Louis, MO, USA) assay was performed similarly to as previously described [51]. After cell treatments, cell culture medium was removed, and the 5 μM DCFH-DA was incubated in PBS for 30 min, at 37 °C and 5% CO_2_. The cell culture plate was washed with PBS, and fluorescence of the cells was read at 485 nm (excitation) and 535 nm (emission) using the multiwall reader Appliskan (Thermo-Fisher Scientific, Vantaa, Finland). Cellular autofluorescence was subtracted as a background using the values of the wells not incubated with the probe.

Similarly, to evaluate reduced GSH levels, monochlorobimane (MCB, Sigma Aldrich, St Louis, MO, USA) assay was performed as previously reported [52]. Cell culture medium was removed, and 50 μM MCB was incubated in PBS for 30 min, at 37 °C and 5% CO_2_. The cells were washed in PBS, and their fluorescence was measured at 355 nm (excitation) and 460 nm (emission).

### 4.7. Cellular Extracts Preparation

Cell extracts were obtained as previously described [53]. Briefly, cells were treated with lysis buffer (20 mM Tris-Cl, pH 7.0; 1% Nonidet P-40; 150 mM NaCl; 10% glycerol; 10 mM EDTA; 20 mM NaF; 5 mM sodium pyrophosphate; and 1 mM Na_3_VO_4_) and freshly added Sigma Aldrich Protease Inhibitor Cocktail and para-Nitrophenylphosphate (pNPP) at 4 °C for 20 min (all from Sigma Aldrich, St Louis, MO, USA). Lysates were sonicated, cleared by centrifugation, and immediately boiled in SDS (Sigma Aldrich, St Louis, MO, USA) reducing sample buffer.

Human HOB nuclei were purified as reported by Casciaro et al. [51]. Briefly, to 5 × 10^6^ cells 400 μL of nuclear isolation buffer (10 mM Tris-HCl, pH 7.8, 1% Nonidet P-40, 10 mM β-mercaptoethanol, 0.5 mM phenylmethylsulfonyl fluoride, 1 μg/mL aprotinin and leupeptin, and 5 mM NaF) was added for 8 min on ice. MilliQ water (400 μL) was then added to swell cells for 3 min. Cells were sheared by passages through a 22-gauge needle. Nuclei were recovered by centrifugation at 400 × *g* at 4 °C for 6 min and washed once in 400 μL of washing buffer (10 mM Tris-HCl, pH 7.4, and 2 mM MgCl_2_, plus inhibitors as described earlier in the text). Supernatants (containing the cytosolic fractions) were further centrifuged for 30 min at 4000× *g*. Isolated nuclear and cytoplasmic extracts were finally lysed in AT lysis buffer, sonicated, and cleared by centrifugation.

### 4.8. SDS-PAGE and Protein Digestion

Nuclear, cytoplasmic, and total lysates were loaded onto 4–16% SDS-PAGE. Electrophoresis was allowed to proceed until proteins were tightly packed in a single band. Gels were then stained in the Coomassie brilliant blue G solution (Sigma Aldrich, St Louis, MO, USA) (0.1% Coomassie blue in 10% acetic acid, 45% methanol) and shaken at room temperature for 1 h. The gels were destained by soaking for 2 h in destaining solution (10% acetic acid, 30% methanol) [29]. In gel trypsin digestion was performed as previously reported [50]. Briefly, each gel band was divided in small pieces that were treated with solution A (1:1 mixture of acetonitrile: 100 mM ammonium bicarbonate) for 30 min and then dried under vacuum. Proteins were then subjected to reduction of disulfide bonds by 10 mM DTT at 56 °C for 1 h. Alkylation of cysteine residues was performed with 55 mM iodoacetamide for 45 min at room temperature in the dark (all from Sigma Aldrich, St Louis, MO, USA). Before trypsin digestion, the rehydration and dehydration steps were again performed with solution A and samples were finally dried under vacuum. Digestion was performed by incubating the dry gel slices with 40 µL of sequencing grade modified trypsin (12.5 ng/µL in 50 mM NH_4_HCO_3_, Sigma Aldrich, St Louis, MO, USA) at 37 °C, overnight. Peptides were extracted from the gels with 100 µL of acetonitrile/0.1% formic acid (3 times) and samples were dried under vacuum and stored at −20 °C until LC-MS/MS analysis was performed.

### 4.9. Mass Spectrometry and Data Analysis

Each sample was dissolved in 40 µL of water:acetonitrile:formic acid (95:3:2), sonicated, centrifuged, and 35 µL of this solution was injected into a UHPLC system (Ultimate 3000, Dionex—Thermo Fisher Scientific, Waltham, MA, USA) coupled to a Q Exactive mass spectrometer (Thermo Fisher Scientific, Waltham, MA, USA) equipped with a HESI-II ion source. Peptides were loaded into a C18 Hypersil Gold (100 × 2.1 mm ID, 1.9 µm ps) column (Thermo Fisher Scientific, Waltham, MA, USA) and separated using a linear gradient of 0.1% formic acid in water (A) and acetonitrile (B) from 2% B to 28% B in 90 min. The mass spectrometer was operated with a Data Dependent Acquisition (DDA) method, performing a 250 <m/z <2000 Full MS scan at 70,000 resolution (at m/z 200) followed by HCD fragmentation at 28 normalized collision energy of the Top 8 most intense precursor ions (charge state z ≥2, 17,500 resolution (at m/z 200)), with a dynamic exclusion of 8 s.

Raw data files were converted to mascot generic format (.mgf) using MSConvert and protein identification was performed using Mascot Server (Version 2.7.0) search engine against the Human section of the neXtProt database [54] and against a database of contaminants commonly found in proteomics experiments (cRAP). The search parameters were set as follows: Trypsin was selected as enzyme with one missed cleavage allowed; carbamidomethylation (C) was specified as fixed modification while oxidation (M) and deamidated (NQ) as variable modifications; peptide and MS/MS tolerances were 10 ppm and 0.02 Da, respectively. False discovery rate (FDR) evaluation was performed using DECOY search tool and results were filtered at 1% FDR for peptide-spectrum matches (PSMs) above homology. Only proteins identified with at least 2 independent peptides were considered as a significant hit. The parameter “Intensity” calculated by the software and normalized based on the total amount of emPAI (exponentially modified Protein Abundance Index) [55] was used to estimate the abundance of the proteins across the different samples. The protein ratio between the EV HOB and HOB samples was then calculated. Only ratio values ≥ 2.0 or ≤ 0.5 were considered. For the Galectin-3, whose ratio was not ≥ 2.0 or ≤ 0.5, the two highest scoring peptides ion peak areas were used to evaluate protein fold-change between EV HOB and HOB samples [56], in order to have a better quantification.

### 4.10. SDS PAGE and Western Blot

Nuclear, cytoplasmic, and whole cell lysates from HOB and AFSC-EV were processed as previously described [21]. Primary antibodies, prepared as previously reported [57], were raised against the following molecules: Actin (Sigma-Aldrich, St Louis, MO, USA), Akt tot, OCN, OPN, osterix, SOD1, SIRT1, TrxR1, Heme Oxygenase 1, MAP LC3β, PARP, IDO, HGF, FKHRL1 FOXO3A, Lamin A/C (Santa Cruz Biotechnology, CA, USA), Rab5 (Lonza, SC, USA), CD9 (Life Technologies, CA, USA), Bcl-2 (Bio Source, CA, USA), p70, p-p70, caspase-7, Galectin-3, p21, pSer473Akt (Cell Signaling Technology, Lieden, Netherlands), Nrf2 (Abcam, Cambridge, UK). Secondary antibodies, used at 1:3000 dilution, were all from Thermo Fisher Scientific (Waltham, MA, USA).

### 4.11. Immunofluorescence and Confocal Microscopy

For immunofluorescence analysis, HOB seeded on coated coverslips were processed and confocal imaging was performed using a Nikon A1 confocal laser scanning microscope, as previously described [58]. Primary antibodies to detect osterix, OCN, 53BP1 (Santa Cruz Biotechnology, CA, USA), p21 (Cell Signaling Technology, Lieden, Netherlands), and pH2A (Millipore, CA, USA) were used following datasheet recommended dilutions. Alexa secondary antibodies (Thermo Fisher Scientific, Waltham, MA, USA) were used at 1:200 dilution. The confocal serial sections were processed with ImageJ software to obtain three-dimensional projections. The image rendering was performed by Adobe Photoshop software.

For apoptosis detection, after 3 washes with 50 μL of binding buffer (10 mM HEPES, pH 7.5, containing 140 mM NaCl and 2.5 mM CaCl_2_), the specimen was incubated for 15 min with 50 μL of double staining solution (binding buffer containing 0.25 μL of annexin V-FITC and 0.25 μL of propidium iodide (PI); BD PharmingenTM, Erembodegem, Belgium). Finally, the specimen was washed 5 times with 50 μL of binding buffer, mounted with 15 μL of binding buffer, and visualized under fluorescence microscopy.

### 4.12. Alizarin Red S Staining

Fixed monolayer cells were washed with distilled water and then incubated with a 2% of Alizarin Red S solution (Sigma Aldrich, St Louis, MO, USA) at pH 4.2 for 30 min at RT. Images of histological samples were obtained with a Zeiss Axiophot microscope (Zeiss AG, Jena, Germany), equipped with a Nikon DS-5Mc CCD color camera.

### 4.13. ALP Assay

Conditioned media were collected and then centrifuged at 20 °C 1200 rpm for 10 min in order to remove cellular debris. Cell lysates were washed twice with cold PBS and next cells were resuspended in 100 µL of Assay Buffer. Samples were homogenized on ice and then centrifuged at 4 °C at 10000 rpm for 15 min to remove any insoluble material. Quantification by Bradford method was performed. Lysates and conditioned media were added in a black 96-well plate, 4 replicates for each condition, and Alkaline Phophatase assay was performed according to the manufacturer’s protocol of ALP assay kit (colorimetric) (Abcam, Cambridge, UK).

### 4.14. Statistical Analysis

Experiments were performed in triplicate. For quantitative comparisons, values were reported as mean ± SD based on triplicate analysis for each sample. To test the significance of observed differences among the study groups One-way ANOVA with Bonferroni post hoc test was applied. A *p* value < 0.05 was considered to be statistically significant. Statistical analysis and plot layout were obtained by using GraphPad Prism^®^ release 6.0 software.

## Supplementary Materials

The following are available online at https://www.mdpi.com/1422-0067/22/1/38/s1.

## Abbreviations

Amniotic fluid stem cells (AFSCs); alkaline phosphatase (ALP); Bovine serum albumin (BSA); 4’,6-diamidino-2-phenylindole (DAPI); dexamethasone (DEXA); dichlorodihydrofluorescein diacetate (DCFH-DA); ethylenediaminetetraacetic acid (EDTA); extracellular vesicles (EV); reduced glutathione (GSH); immunofluorescence (IF); phosphate buffered saline (PBS); 4-(2-hydroxyethyl)-1-piperazineethanesulfonic acid (HEPES); monochlorobimane (MCB); sodium dodecyl sulfate (SDS); western blot (WB).

## References

[1] Hennemann B.A. (2002). Osteoporose: Prävention, diagnose und therapie. Med. Mon. Pharm..

[2] Raisz L.G. (2005). Pathogenesis of osteoporosis: Concepts, conflicts, and prospects. J. Clin. Investig..

[3] Owen R., Reilly G.C. (2018). In vitro Models of Bone Remodelling and Associated Disorders. Front. Bioeng. Biotechnol..

[4] Zhang X., Chen K., Wei B., Liu X., Lei Z., Bai X. (2016). Ginsenosides Rg3 attenuates glucocorticoid-induced osteoporosis through regulating BMP-2/BMPR1A/Runx2 signaling pathway. Chem. Biol. Interact..

[5] Liu Y., Porta A., Peng X., Gengaro K., Cunningham E.B., Li H., Dominguez L.A., Bellido T., Christakos S. (2004). Prevention of glucocorticoid-induced apoptosis in osteocytes and osteoblasts by calbindin-D28k. J. Bone Miner. Res..

[6] Palumbo C., Ferretti M., Ardizzoni A., Zaffe D., Marotti G. (2001). Perspective Article Osteocyte-osteoclast morphological relationships and the putative role of osteocytes in bone remodeling. J. Musculoskelet. Neuronal Interact..

[7] Rodriguez J.P., Montecinos L., Ros S., Reyes P., Martnez J. (2000). Mesenchymal stem cells from osteoporotic patients produce a type I collagen-deficient extracellular matrix favoring adipogenic differentiation. J. Cell. Biochem..

[8] Liang M., Liu W., Peng Z., Lv S., Guan Y., An G., Zhang Y., Huang T., Wang Y. (2019). The therapeutic effect of secretome from human umbilical cord-derived mesenchymal stem cells in age-related osteoporosis. Artif. Cells Nanomed. Biotechnol..

[9] Li Y., Jin D., Xie W., Wen L., Chen W., Xu J., Ding J., Ren D., Xiao Z. (2018). Mesenchymal Stem Cells-Derived Exosomes: A Possible Therapeutic Strategy for Osteoporosis. Curr. Stem Cell Res. Ther..

[10] Tan S.H.S., Wong J.R.Y., Sim S.J.Y., Tjio C.K.E., Wong K.L., Chew J.R.J., Hui J.H.P., Toh W.S. (2020). Mesenchymal stem cell exosomes in bone regenerative strategies—A systematic review of preclinical studies. Mater. Today Bio.

[11] Liu S., Xu X., Liang S., Chen Z., Zhang Y., Qian A., Hu L. (2020). The Application of MSCs-Derived Extracellular Vesicles in Bone Disorders: Novel Cell-Free Therapeutic Strategy. Front. Cell Dev. Biol..

[12] Zhao P., Xiao L., Peng J., Qian Y.Q., Huang C.C. (2018). Exosomes derived from bone marrow mesenchymal stem cells improve osteoporosis through promoting osteoblast proliferation via MAPK pathway. Eur. Rev. Med. Pharmacol. Sci..

[13] Ren L., Song Z.J., Cai Q.W., Chen R.X., Zou Y., Fu Q., Ma Y.Y. (2019). Adipose mesenchymal stem cell-derived exosomes ameliorate hypoxia/serum deprivation-induced osteocyte apoptosis and osteocyte-mediated osteoclastogenesis in vitro. Biochem. Biophys. Res. Commun..

[14] Qi X., Zhang J., Yuan H., Xu Z., Li Q., Niu X., Hu B., Wang Y., Li X. (2016). Exosomes secreted by human-induced pluripotent stem cell-derived mesenchymal stem cells repair critical-sized bone defects through enhanced angiogenesis and osteogenesis in osteoporotic rats. Int. J. Biol. Sci..

[15] Sonoda S., Murata S., Nishida K., Kato H., Uehara N., Kyumoto Y.N., Yamaza H., Takahashi I., Kukita T., Yamaza T. (2020). Extracellular vesicles from deciduous pulp stem cells recover bone loss by regulating telomerase activity in an osteoporosis mouse model. Stem Cell Res. Ther..

[16] Hu Y., Xu R., Chen C.Y., Rao S.S., Xia K., Huang J., Yin H., Wang Z.X., Cao J., Liu Z.Z. (2019). Extracellular vesicles from human umbilical cord blood ameliorate bone loss in senile osteoporotic mice. Metab. Clin. Exp..

[17] Kobayashi K., Nojiri H., Saita Y., Morikawa D., Ozawa Y., Watanabe K., Koike M., Asou Y., Shirasawa T., Yokote K. (2015). Mitochondrial superoxide in osteocytes perturbs canalicular networks in the setting of age-related osteoporosis. Sci. Rep..

[18] Deng S., Dai G., Chen S., Nie Z., Zhou J., Fang H., Peng H. (2019). Dexamethasone induces osteoblast apoptosis through ROS-PI3K/AKT/GSK3β signaling pathway. Biomed. Pharmacother..

[19] Yang X., Jiang T., Wang Y., Guo L. (2019). The Role and Mechanism of SIRT1 in Resveratrol-regulated Osteoblast Autophagy in Osteoporosis Rats. Sci. Rep..

[20] Pierrefite-Carle V., Santucci-Darmanin S., Breuil V., Camuzard O., Carle G.F. (2015). Autophagy in bone: Self-eating to stay in balance. Ageing Res. Rev..

[21] Zavatti M., Beretti F., Casciaro F., Bertucci E., Maraldi T. (2019). Comparison of the therapeutic effect of amniotic fluid stem cells and their exosomes on monoiodoacetate-induced animal model of osteoarthritis. BioFactors.

[22] Gatti M., Zavatti M., Beretti F., Giuliani D., Vandini E., Ottani A., Bertucci E., Maraldi T. (2020). Oxidative Stress in Alzheimer’s Disease: In Vitro Therapeutic Effect of Amniotic Fluid Stem Cells Extracellular Vesicles. Oxidative Med. Cell. Longev..

[23] Kubatzky K.F., Uhle F., Eigenbrod T. (2018). From macrophage to osteoclast—How metabolism determines function and activity. Cytokine.

[24] Vousden K.H. (2006). Outcomes of p53 activation—Spoilt for choice. J. Cell Sci..

[25] Huang R., Xu Y., Wan W., Shou X., Qian J., You Z., Liu B., Chang C., Zhou T., Lippincott-Schwartz J. (2015). Deacetylation of nuclear LC3 drives autophagy initiation under starvation. Mol. Cell.

[26] Chen W., Sun Z., Wang X.J., Jiang T., Huang Z., Fang D., Zhang D.D. (2009). Direct Interaction between Nrf2 and p21Cip1/WAF1 Upregulates the Nrf2-Mediated Antioxidant Response. Mol. Cell.

[27] Jensen E.D., Gopalakrishnan R., Westendorf J.J. (2010). Regulation of gene expression in osteoblasts. BioFactors.

[28] Huang W., Yang S., Shao J., Li Y.P. (2007). Signaling and transcriptional regulation in osteoblast commitment and differentiation. Front. Biosci..

[29] Beretti F., Zavatti M., Casciaro F., Comitini G., Franchi F., Barbieri V., La Sala G.B., Maraldi T. (2018). Amniotic fluid stem cell exosomes: Therapeutic perspective. BioFactors.

[30] Iacobini C., Fantauzzi C.B., Pugliese G., Menini S. (2017). Role of galectin-3 in bone cell differentiation, bone pathophysiology and vascular osteogenesis. Int. J. Mol. Sci..

[31] Li D.Y., Yu J.C., Xiao L., Miao W., Ji K., Wang S.C., Geng Y.X. (2017). Autophagy attenuates the oxidative stress-induced apoptosis of Mc3T3-E1 osteoblasts. Eur. Rev. Med. Pharmacol. Sci..

[32] Zhang S., Liu Y., Liang Q. (2018). Low-dose dexamethasone affects osteoblast viability by inducing autophagy via intracellular ros. Mol. Med. Rep..

[33] De Godoy M.A., Saraiva L.M., de Carvalho L.R.P., Vasconcelos-dos-Santos A., Beiral H.J.V., Ramos A.B., de Paula Silva L.R., Leal R.B., Monteiro V.H.S., Braga C.V. (2018). Mesenchymal stem cells and cell-derived extracellular vesicles protect hippocampal neurons from oxidative stress and synapse damage induced by amyloid- oligomers. J. Biol. Chem..

[34] Almeida M., Porter R.M. (2019). Sirtuins and FoxOs in osteoporosis and osteoarthritis. Bone.

[35] Li X., Xu J., Dai B., Wang X., Guo Q., Qin L. (2020). Targeting autophagy in osteoporosis: From pathophysiology to potential therapy. Ageing Res. Rev..

[36] Wen J., Fang F., Guo S.H., Zhang Y., Peng X.L., Sun W.M., Wei X.R., He J.S., Hung T. (2018). Amyloid β-Derived Diffusible Ligands (ADDLs) Induce Abnormal Autophagy Associated with Aβ Aggregation Degree. J. Mol. Neurosci..

[37] Huang K., Chen C., Hao J., Huang J., Wang S., Liu P., Huang H. (2015). Polydatin promotes Nrf2-ARE anti-oxidative pathway through activating Sirt1 to resist AGEs-induced upregulation of fibronetin and transforming growth factor-β1 in rat glomerular messangial cells. Mol. Cell. Endocrinol..

[38] Hauck L., Harms C., Grothe D., An J., Gertz K., Kronenberg G., Dietz R., Endres M., Von Harsdorf R. (2007). Critical role for FoxO3a-dependent regulation of p21CIP1/WAF1 in response to statin signaling in cardiac myocytes. Circ. Res..

[39] Brunet A., Sweeney L.B., Sturgill J.F., Chua K.F., Greer P.L., Lin Y., Tran H., Ross S.E., Mostoslavsy R., Cohen H.Y. (2004). Stress-Dependent Regulation of FOXO Transcription Factors by the SIRT1 Deacetylase. Science.

[40] Gorospe M., Wang X., Holbrook N.J. (1999). Functional role of p21 during the cellular response to stress. Gene Expr. J. Liver Res..

[41] Ward I.M., Minn K., Jorda K.G., Chen J. (2003). Accumulation of checkpoint protein 53BP1 at DNA breaks involves its binding to phosphorylated histone H2AX. J. Biol. Chem..

[42] Flores E.R., Tsai K.Y., Crowley D., Sengupta S., Yang A., McKeon F., Jack T. (2002). p63 and p73 are required for p53-dependent apoptosis in response to DNA damage. Nature.

[43] Napoli M., Flores E.R. (2013). The family that eats together stays together: New p53 family transcriptional targets in autophagy. Genes Dev..

[44] Cecchinelli B., Lavra L., Rinaldo C., Iacovelli S., Gurtner A., Gasbarri A., Ulivieri A., Del Prete F., Trovato M., Piaggio G. (2006). Repression of the Antiapoptotic Molecule Galectin-3 by Homeodomain-Interacting Protein Kinase 2-Activated p53 Is Required for p53-Induced Apoptosis. Mol. Cell. Biol..

[45] Fritsch K., Mernberger M., Nist A., Stiewe T., Brehm A., Jacob R. (2016). Galectin-3 interacts with components of the nuclear ribonucleoprotein complex. BMC Cancer.

[46] Mercer N., Ahmed H., McCarthy A.D., Etcheverry S.B., Vasta G.R., Cortizo A.M. (2004). AGE-R3/galectin-3 expression in osteoblast-like cells: Regulation by AGEs. Mol. Cell. Biochem..

[47] Kawamata A., Izu Y., Yokoyama H., Amagasa T., Wagner E.F., Nakashima K., Ezura Y., Hayata T., Noda M. (2008). JunD suppresses bone formation and contributes to low bone mass induced by estrogen depletion. J. Cell. Biochem..

[48] Wu J., Yang Y., He Y., Li Q., Wang X., Sun C., Wang L., An Y., Luo F. (2019). EFTUD2 gene deficiency disrupts osteoblast maturation and inhibits chondrocyte differentiation via activation of the p53 signaling pathway. Hum. Genom..

[49] Zavatti M., Beretti F., Casciaro F., Comitini G., Franchi F., Barbieri V., Bertoni L., De Pol A., La Sala G.B., Maraldi T. (2017). Development of a novel method for amniotic fluid stem cell storage. Cytotherapy.

[50] Maraldi T., Beretti F., Anselmi L., Franchin C., Arrigoni G., Braglia L., Mandrioli J., Vinceti M., Marmiroli S. (2019). Influence of selenium on the emergence of neuro tubule defects in a neuron-like cell line and its implications for amyotrophic lateral sclerosis. NeuroToxicology.

[51] Casciaro F., Beretti F., Zavatti M., McCubrey J.A., Ratti S., Marmiroli S., Follo M.Y., Maraldi T. (2018). Nuclear Nox4 interaction with prelamin A is associated with nuclear redox control of stem cell aging. Aging.

[52] Marrazzo P., Angeloni C., Freschi M., Lorenzini A., Prata C., Maraldi T., Hrelia S. (2018). Combination of epigallocatechin gallate and sulforaphane counteracts in vitro oxidative stress and delays stemness loss of amniotic fluid stem cells. Oxidative Med. Cell. Longev..

[53] Casciaro F., Borghesan M., Beretti F., Zavatti M., Bertucci E., Follo M.Y., Maraldi T., Demaria M. (2020). Prolonged hypoxia delays aging and preserves functionality of human amniotic fluid stem cells. Mech. Ageing Dev..

[54] Zahn-Zabal M., Michel P.A., Gateau A., Nikitin F., Schaeffer M., Audot E., Gaudet P., Duek P.D., Teixeira D., De Laval V.R. (2020). The neXtProt knowledgebase in 2020: Data, tools and usability improvements. Nucleic Acids Res..

[55] Arike L., Peil L. (2014). Spectral Counting Label-Free Proteomics. Methods Mol. Biol..

[56] Old W.M., Meyer-Arendt K., Aveline-Wolf L., Pierce K.G., Mendoza A., Sevinsky J.R., Resing K.A., Ahn N.G. (2005). Comparison of label-free methods for quantifying human proteins by shotgun proteomics. Mol. Cell. Proteom..

[57] Naeem A.S., Zhu Y., Di W.L., Marmiroli S., O’Shaughnessy R.F. (2015). AKT1-mediated Lamin A/C degradation is required for nuclear degradation and normal epidermal terminal differentiation. Cell Death Differ..

[58] Prata C., Facchini C., Leoncini E., Lenzi M., Maraldi T., Angeloni C., Zambonin L., Hrelia S., Fiorentini D. (2018). Sulforaphane modulates AQP8-linked redox signalling in leukemia cells. Oxidative Med. Cell. Longev..

