# Supplementary material for: Amniotic Fluid Stem Cell-Derived Extracellular Vesicles Counteract Steroid-Induced Osteoporosis In Vitro
Source: Int J Mol Sci. 2020 Dec 22;22(1):38. doi: 10.3390/ijms22010038 (PMC7792960; doi:10.3390/ijms22010038)
Supplement: Supplementary file 1 [file ijms-22-00038-s001.pdf]

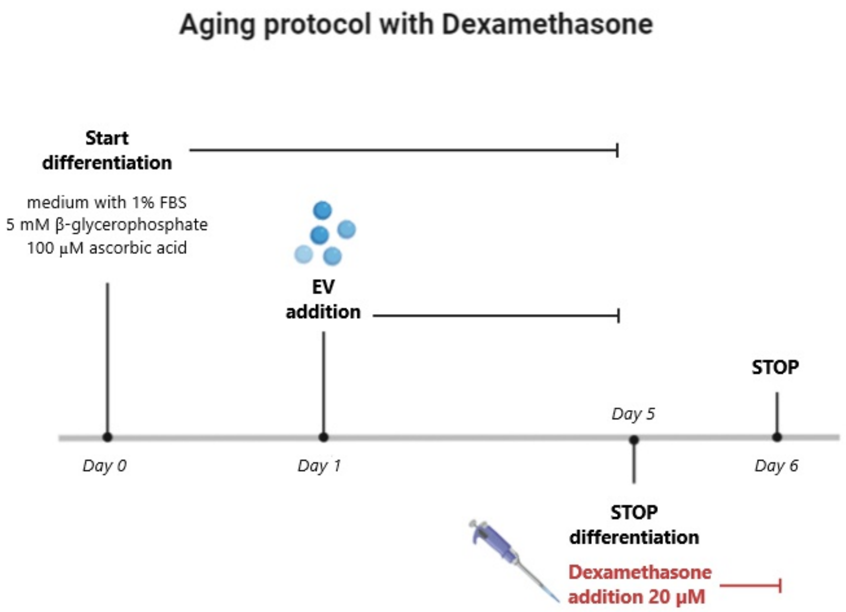

Supplementary Figure 1
Experimental scheme - HOB cells were exposed to the differentiation medium at day 0, the day after EV were added until day 5. Finally, cells were exposed to Dexa for 24 h.

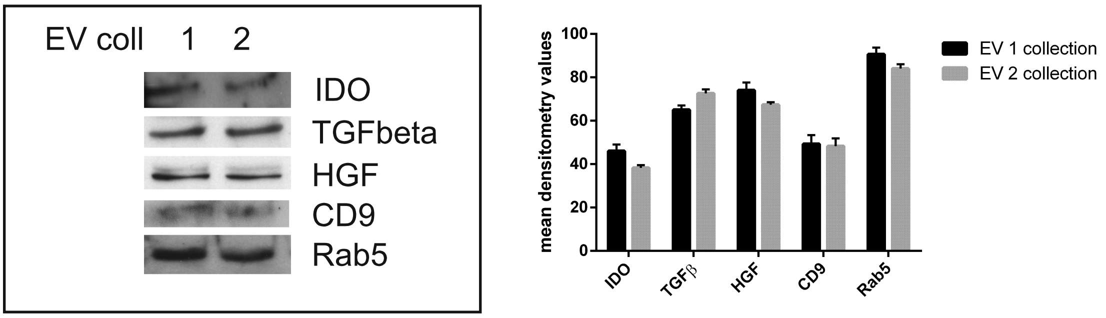

Supplementary Figure 2

Representative WB of EV obtained from a first (1) and a second (2) collection. Images and related quantitative graph of the presence of the following proteins: IDO, TGFbeta, HGF, as immunomodulating factors, and CD9 and Rab5, as EV markers.
